# Thermodynamic Additivity of Sequence Variations: An Algorithm for Creating High Affinity Peptides Without Large Libraries or Structural Information

**DOI:** 10.1371/journal.pone.0015432

**Published:** 2010-11-11

**Authors:** Matthew P. Greving, Paul E. Belcher, Chris W. Diehnelt, Maria J. Gonzalez-Moa, Jack Emery, Jinglin Fu, Stephen Albert Johnston, Neal W. Woodbury

**Affiliations:** 1 Center for BioOptical Nanotechnology and Center for Innovations in Medicine, The Biodesign Institute, Arizona State University, Tempe, Arizona, United States of America; 2 School of Life Sciences, Arizona State University, Tempe, Arizona, United States of America; 3 Department of Chemistry and Biochemistry, Arizona State University, Tempe, Arizona, United States of America; Indiana University School of Medicine, United States of America

## Abstract

**Background:**

There is a significant need for affinity reagents with high target affinity/specificity that can be developed rapidly and inexpensively. Existing affinity reagent development approaches, including protein mutagenesis, directed evolution, and fragment-based design utilize large libraries and/or require structural information thereby adding time and expense. Until now, no systematic approach to affinity reagent development existed that could produce nanomolar affinity from small chemically synthesized peptide libraries without the aid of structural information.

**Methodology/Principal Findings:**

Based on the principle of additivity, we have developed an algorithm for generating high affinity peptide ligands. In this algorithm, point-variations in a lead sequence are screened and combined in a systematic manner to achieve additive binding energies. To demonstrate this approach, low-affinity lead peptides for multiple protein targets were identified from sparse random sequence space and optimized to high affinity in just two chemical steps. In one example, a TNF-α binding peptide with K_d_ = 90 nM and high target specificity was generated. The changes in binding energy associated with each variation were generally additive upon combining variations, validating the basis of the algorithm. Interestingly, cooperativity between point-variations was not observed, and in a few specific cases, combinations were less than energetically additive.

**Conclusions/Significance:**

By using this additivity algorithm, peptide ligands with high affinity for protein targets were generated. With this algorithm, one of the highest affinity TNF-α binding peptides reported to date was produced. Most importantly, high affinity was achieved from small, chemically-synthesized libraries without the need for structural information at any time during the process. This is significantly different than protein mutagenesis, directed evolution, or fragment-based design approaches, which rely on large libraries and/or structural guidance. With this algorithm, high affinity/specificity peptide ligands can be developed rapidly, inexpensively, and in an entirely chemical manner.

## Introduction

A comprehensive survey of the human proteome requires a vast library of specfic affinity reagents [1]–[4]. Building such a library requires strategies that are high-throughput, inexpensive and have the flexibility to produce ligands that are compatible with numerous applications such as gel-based analysis, histology, microarrays, purification columns, etc. [4]. Several well-established approaches have proven to be a rich source of affinity reagents. These include *in vitro* selection of peptides [5]–[7], protein mutagenesis [8], computational design [9]–[12], aptamer selection [13], bead-based library screens [14], and fragment-based design of small molecules [3]. While these approaches generally produce high-quality affinity reagents, they don't fully meet the need for rapid development, low cost, and application-specific flexibility. The utilization of large chemical libraries during several stages of affinity reagent development and/or the need for structural information extend the completion time, increase the cost, and limit the flexibility with these approaches [3], [8], [15], [16]. A new approach that addresses these points would have the potential to serve the need for large numbers of inexpensive affinity reagents for proteomics.

Driven by this need, we have explored a fundamentally different, systematic strategy to develop high affinity binders to a given protein target. Our guiding hypothesis was that weak binding lead peptides could be readily identified from a screen of a small chemical library of unstructured short random peptide sequences. Once identified, these leads could then be systematically improved to high affinity by utilizing thermodynamic additivity of sequence variations. If successful, such an algorithm would be generally applicable and enable high-throughput, low-cost production of peptide affinity reagents with significant flexibility due to the fact that the process is based solely on chemical synthesis of small libraries, rather than large enzymatically generated libraries. Importantly, because the algorithm relies on additive interactions, it does not require structural knowledge about the target. This is a principal characteristic that differentiates this algorithm from other approaches that try to identify cooperative interactions, which frequently require structural information about the target protein and possibly the affinity reagent/target complex [3], [9], thereby significantly reducing throughput and increasing cost. Here, we test the concepts of this algorithm by generating high affinity peptide ligands to protein targets.

In addition to the practical aspects of such an algorithm, the effects of sequence perturbation(s) on protein-peptide binding thermodynamics and specificity with short peptides derived from random chemical libraries remains largely unexplored. Protein-protein and protein-small molecule binding thermodynamics have been extensively studied [17]–[21], but due to their highly structured nature, it is not obvious that the same energetic behavior observed in these complexes can be expected with short unstructured peptides. In a protein-protein or protein-small molecule complex, the overall binding energy can be an additive or non-additive accumulation of component free energies, or a combination of additive and non-additive effects. Additivity of component free energies is observed when the component interactions do not structurally interact and contribute independently to the standard free energy of binding [22]. Conversely, non-additive cooperative contributions to binding free energy are observed when individual components are structurally connected. The work presented here provides insight into the additive/non-additive protein binding thermodynamics and specificity of sequence variations in short unstructured peptide ligands identified from random chemically-synthesized sequence space.

## Results

**Figure 1:**
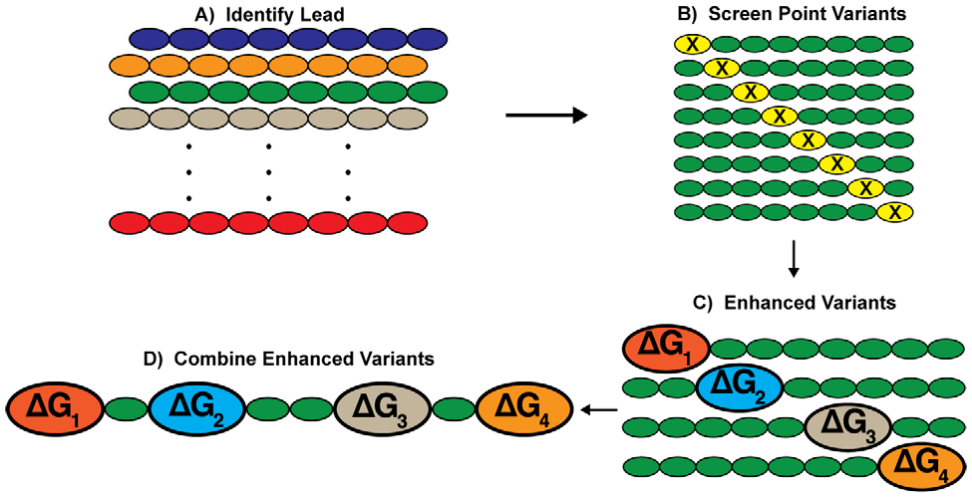
Schematic of the additivity algorithm. A) A lead sequence is first identified from a peptide library; this algorithm is not dependent on the source of the initial library (i.e. chemical library, *in vitro* library, etc.). B) A small library of point-variants (X =  any substitution) is synthesized to C) identify substitutions that enhance target affinity. D) Enhanced point-variants are combined to produce a peptide with a relative binding free energy approximately equal to the sum of the component energies.

The basic strategy is illustrated in Figure 1. We choose to test this strategy by creating ligands to the cytokine, tumor-necrosis factor alpha (TNF-α) [23]–[25]. TNF-α is a high-value target for which several antibody and small molecule therapeutics have been developed [26], [27]. TNF-α affinity reagents have been isolated by other procedures [28]–[30], and this is useful for comparison. If successful, a ligand for TNF-α could be used as an affinity reagent by itself or as a component of a multivalent ligand [31].

### Lead Generation from a Sparse Random Library

A library of 10^4^ random 20-mer peptides (17 randomized positions, 3 fixed positions at the C-terminus) was screened in order to generate lead peptides with affinity for TNF-α. 20-mer peptides were chosen because they are not expected to adopt stable structures that would interfere with the ability of different residues in the peptide to bind independently (Figures S1-3 in File S1). Preliminary microarray experiments showed that this library contains sufficient chemical diversity to identify lead peptides with measurable target binding affinity and specificity (CWD and SAJ unpublished data). This is likely possible because only a few favorable contacts between a peptide and a target protein are needed for observable binding [32].

Each member of this library of 10^4^ peptides was individually screened by surface plasmon resonance (SPR) and 171 peptide sequences were identified as potential leads with affinity for TNF-α. The significant number of potential lead sequences allowed for the application of more stringent lead criteria such as good solubility, high peptide sample purity and low background binding to the SPR chip surface. To minimize non-specific binding, the number of potential leads was further reduced by comparing TNF-α SPR binding response to a panel of four unrelated proteins (AKT1, Neutravidin, Transferrin, and Ubiquitin). Two peptides, FERDPLMMPWSFLQSRQGSC (referred to as TNF1, note that the GSC sequence at the end was common to all peptides in the library) and YGPSDAFKITRFHQQSSGSC (referred to as TNF4) were chosen as lead peptides for optimization based upon: their respective dissociation constants (K_d_) of 160±19 µM and 23±3.5 µM for TNF-α; their minimal off-target binding (Figure S4 in File S1); and their relative solubility indicated by average hydropathy (GRAVY)[33] scores of −0.52 and −0.77, respectively.

### Point-Variant Scanning of the TNF1 Lead Peptide

A small library of TNF1 point-variants was generated to identify higher affinity peptides. For a 20-residue peptide, it is feasible to synthesize many, if not all, of the possible point-variants by standard commercial synthesis and screen them individually. In this study, only a subset of amino acids was substituted at each position in TNF1, making the number of peptides in the library more manageable. The specific amino acids used were selected based on data from previous protein interaction studies [34]–[38]. A library of TNF1 point-variants containing all substitutions of the amino acid set {Y, A, D, S, K, N, V, W} in each of the 17 randomized positions (132 unique point-variants) was synthesized. Tyrosine (Y), alanine (A), aspartic acid (D) and serine (S) were selected because of their effectiveness in producing high affinity interactions when substituted into the complementary-determining regions (CDRs) of synthetic antibodies [34], lysine (K) was selected to balance the charge in the substitution set, asparagine (N), valine (V) and tryptophan (W) were selected to span the hydropathy range [33]. This set of 132 point-variants was screened for relative TNF-α SPR binding response using a 50 µM peptide concentration in each case. This concentration is approximately 3-fold below the K_d_ of TNF1, and was used in order to increase the high-end dynamic range for quantifying enhancing point variations.

**Figure 2:**
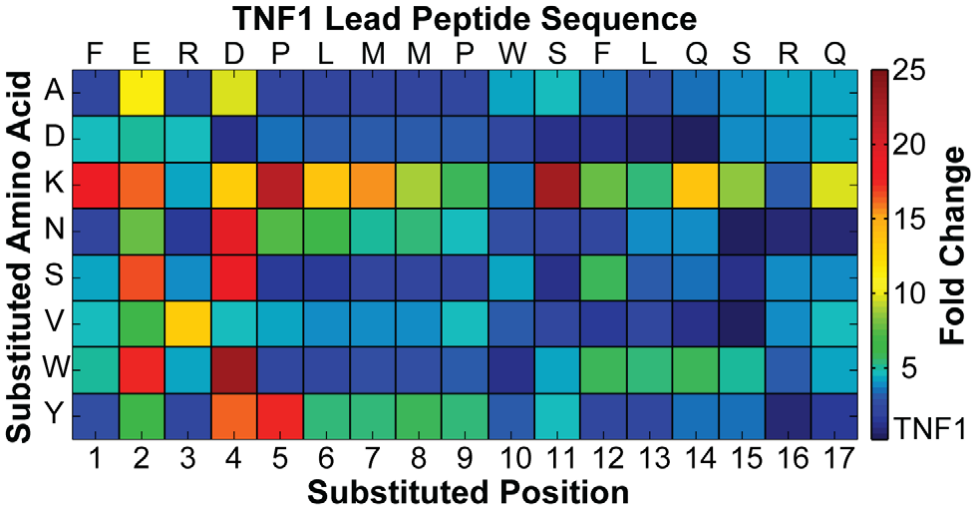
Fold-change heat map from the SPR screen of TNF1 point-variants. Fold-change relative to the TNF1 lead peptide was calculated from an average binding response after a 60-second association across several replicate injections of a fixed 50 µM peptide concentration. Peptides were assayed at a concentration well below the dissociation constant of the lead peptide (K_d_ ∼160 µM) to improve the high-end dynamic range of responses. Point variation nomenclature: ‘P6Y’, indicates the original proline in position 6 is substituted with a tyrosine in the corresponding variant peptide.

The results from the TNF1 point-variant screen are represented as a heat map (Figure 2). The heat map reveals that variations at 9 unique positions in the sequence result in a greater than 10-fold increase in the SPR binding response relative to TNF1. Most notably, the heat map suggests that negative charges in the lead peptide may decrease TNF-α binding; almost any variation in position 2 (E) or 4 (D) enhances affinity, including alanine, which is considered a neutral substitution in scanning mutagenesis [39]. Further support for the importance of the overall charge of the peptide comes from the fact that substituting lysine in several positions enhances affinity. This suggests that the optimized peptide should have a higher pI than TNF1. In addition to the effects of negative charge, the heat map indicates that tyrosine is a particularly favorable substitution in the N-terminal half of the peptide. Tyrosine is the most favorable uncharged substitution in the point-variant library, with 7 out of the 17 positions substituted with tyrosine producing better than 5-fold enhancement. This is in agreement with protein mutagenesis studies that show tyrosine to be the most effective amino acid for producing favorable protein-protein interactions [34]–[38]. Also, affinity enhancement from tyrosine or lysine substitution in several positions of the peptide is consistent with the idea that, in mostly unstructured peptides, modest affinity can be achieved from a few interacting residues separated by relatively flexible non-interacting residues.

Five of the affinity-increasing point-variants (D4S, D4Y, P5Y, M7K, S11K) were selected for further characterization because they showed a ≥15-fold enhancement in SPR binding response relative to TNF1 as well as low non-specific binding on the SPR chip. TNF-α affinities (K_d_) for the selected point-variant sequences were determined by equilibrium SPR measurements (Table 1). Consistent with the initial point-variant screen, the selected variants have significantly higher affinity than TNF1, exhibiting an average 3.4 fold K_d_ improvement.

**Table 1:** TNF1 lead and point-variant binding energies and affinities.^a^

Peptide	TNF1 Lead	D4S	D4Y	P5Y	M7K	S11K
**Standard Binding ΔG° (kcal/mol)**	−5.21±0.07	−5.98±0.04	−5.95±0.06	−5.79±0.04	−5.93±0.20	−6.03±0.10
**K_d_ (µM)**	**160±19**	**42±2.4**	**44±4.8**	**58±3.4**	**57±20**	**40±7.2**
**K_d_ Fold-Change Relative to Lead**	-	3.8±0.5	3.6±0.6	2.7±0.4	2.8±1.0	3.9±0.9
**Variant Relative ΔG Contribution (kcal/mol)**	-	−0.77±0.08	−0.74±0.10	−0.58±0.08	−0.72±0.22	−0.82±0.13

a Standard binding free energies and dissociation constants (K_d_) were calculated separately as an average of several replicate measurements. Point-variant relative contributions were calculated as the difference between the standard binding free energy of a particular point-variant from that of the lead.

### Affinity Prediction of TNF1 Multiple Variants

Relative binding free energies for each point variation were calculated as the difference between the standard free energy of binding for the point-variant sequence and that of the lead sequence. The resulting relative binding free energies for the D4S, D4Y, P5Y, M7K and S11K variants are given in Table 1. From these individual contributions and the assumption that combining point variations would result in a sum of their relative binding free energies, the binding free energies of variant sequences containing multiple substitutions can be predicted.

Thus, a combination of 4 point variations (D4S+P5Y+M7K+S11K) is predicted to have a K_d_ ∼1 µM, an approximate 100-fold improvement relative to the lead peptide (K_d_ = 160 µM). As a result of these predictions, this quadruple variant, referred to as TNF1-opt, was selected as the optimized sequence. The D4S substitution was selected over the D4Y substitution because a tyrosine substitution in position 5 (P5Y) also showed significant improvement, which suggests there may be a proximity effect of tyrosine substitution in this region of the peptide. In other words, it may be that tyrosine can produce an affinity enhancement in either position 4 or 5 but not both positions, particularly given the large size of tyrosine. Therefore, a serine substitution was used in position 4 (D4S) and tyrosine in position 5 (P5Y). In addition to the TNF1-opt quadruple variant, several intermediate variants (double, triple variants) were characterized to compare predicted affinities to observed TNF-α affinities.

### Affinity Characterization of TNF1 Double, Triple and Quadruple Variants

**Figure 3:**
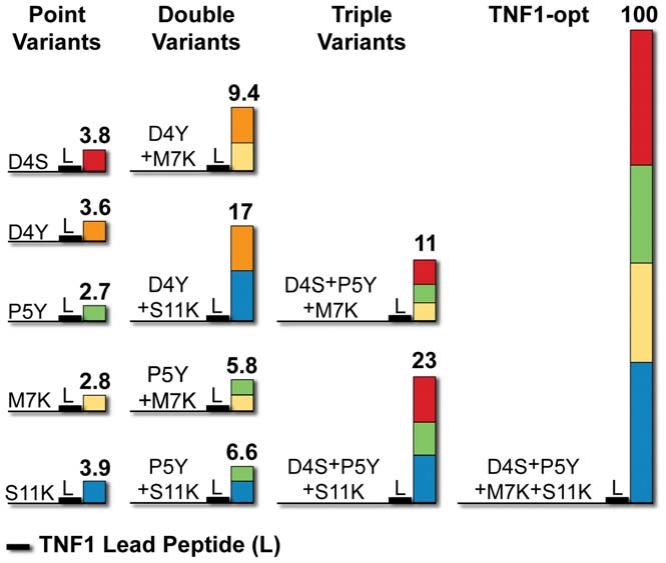
Fold-change in TNF-α affinity across four generations of TNF1 variant sequences. Fold-change relative to TNF1 is above the bars in bold and is calculated from the association constant (K_a_ = 1/K_d_) of a variant divided by the K_a_ of TNF1.

**Figure 4:**
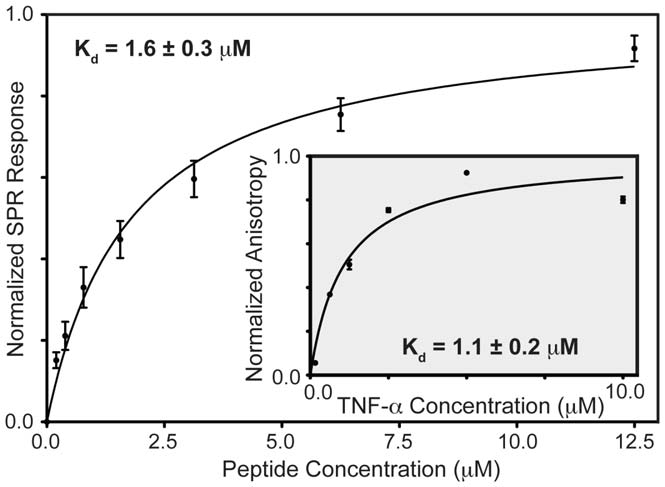
Equilibrium binding isotherms for TNF1-opt. Outer plot: SPR normalized replicate responses performed with TNF-α captured on the SPR chip surface and variable TNF-opt concentrations flowed over the surface. Inset plot: Solution-phase fluorescence anisotropy measurements performed in triplicate with TNF1-opt labeled at the C-terminal cysteine, TNF-opt concentration was fixed at 100 nM while TNF-α concentration was varied. TNF1-opt sensorgrams are available in Figure S5 in File S1, SPR binding isotherms for all enhanced variants tested are available in Figure S6 in File S1.

Four double (D4Y+M7K, D4Y+S11K, P5Y+M7K, P5Y+S11K), two triple (D4S+P5Y+M7K, D4S+P5Y+S11K) and one quadruple (D4S+P5Y+M7K+S11K) variant sequences were characterized with SPR. In all cases, an improvement in TNF-α affinity was observed when an additional enhancing substitution was added to the sequence. Double variants had higher affinities than the corresponding single variants, triple variants had higher affinities than the corresponding single/double variants and the quadruple variant had the greatest affinity (Figure 3, Table 2). The optimized quadruple variant sequence (TNF1-opt) has a K_d_ = 1.6±0.3 µM determined by equilibrium SPR measurements (Figure 4, Figures S5, S6 in File S1). Further validation of TNF1-opt affinity was performed using fluorescence anisotropy, resulting in a K_d_ = 1.1±0.2 µM, in agreement with the affinity determined by SPR (Figure 4).

**Table 2:** TNF1 multiple variant observed/predicted binding energies and affinities.^a^

	Peptide	D4Y+M7K	D4Y+S11K	P5Y+M7K	P5Y+S11K	D4S+P5Y +M7K	D4S+P5Y +S11K	D4S+P5Y +M7K+S11K
**Observed**	**Standard Binding ΔG° (kcal/mol)**	−6.54±0.07	−6.87±0.05	−6.24±0.04	−6.31±0.04	−6.63±0.04	−7.03±0.04	−7.97±0.11
	**K_d_ (µM)**	**17±1.9**	**9.3±0.7**	**27±1.8**	**24±1.4**	**14±1.0**	**7.0±0.5**	**1.6±0.3**
	**K_d_ Fold-Change Relative to Lead**	9.4±1.6	17±2.5	5.8±0.8	6.6±0.9	11±1.6	23±3.2	100±22
**Predicted**	**Standard Binding ΔG° (kcal/mol)**	-6.66±0.25	-6.77±0.18	-6.51±0.24	-6.61±0.17	-7.28±0.26	-7.38±0.19	−8.10±0.29
	**K_d_ Range (µM)**	**20-8.5**	**15-8.0**	**25-11**	**19-11**	**7.0-3.0**	**5.3-2.8**	**1.9-0.7**

a Standard binding free energies and dissociation constants (K_d_) were calculated separately as an average of several replicate measurements. Predicted standard binding free energies were calculated as a sum of the standard binding free energy of the lead and the relative binding free energy contribution of the point-variations (Table 1) substituted into the corresponding multiple variant sequences.

Kinetic fits of the TNF1 and TNF1-opt SPR sensorgrams (Figure S7 in File S1) indicate that TNF1-opt has approximately an order of magnitude or more improvement in both on-rate (k_on_), and off-rate (k_off_), when compared to TNF1 (TNF1: k_off_ = 1.6±0.5 s^−1^, k_on_ = 5.0±1.7×10^3^ M^−1^s^−1^, TNF1-opt: k_off_ = 0.2±0.02 s^−1^ k_on_ = 2.6±0.2×10^5^ M^−1^s^−1^). From these rate constants, one can calculate a K_d_ of 0.70±0.02 µM for TNF1-opt, which is comparable to the affinities determined from equilibrium SPR binding responses and fluorescence anisotropy (Figure 4).

### Comparison of TNF1 Multiple Variant Observed Affinities to Predicted Affinities

**Figure 5:**
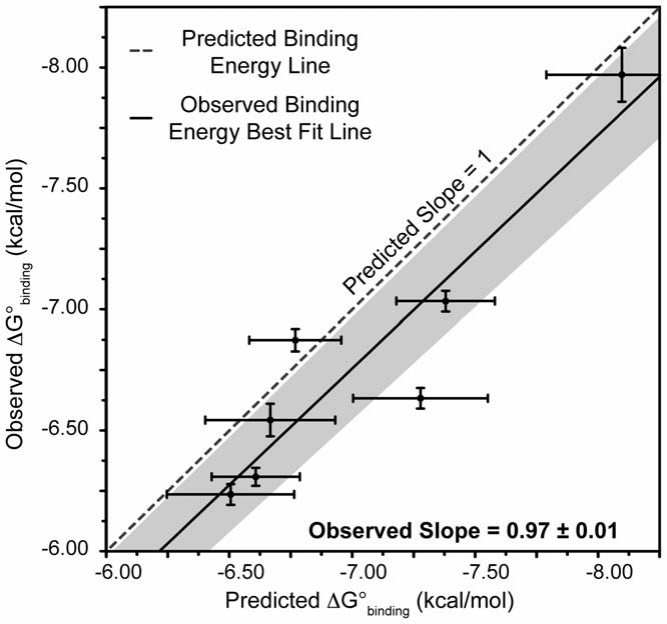
TNF1 multiple variant standard binding free energies: observed vs. predicted assuming thermodynamic additivity. Observed standard binding free energies were calculated from the dissociation constants measured across several replicate experiments, predicted standard binding free energies were calculated as the sum of the standard binding free energy of TNF1 and relative binding free energy contributions from the corresponding point-variants. The 95% confidence interval for the best-fit line (solid line) is shaded. The observed slope (0.97±0.01) of the best-fit line is in good agreement with the slope predicted from thermodynamic additivity (predicted  = 1).

When the standard free energy of binding determined from the K_d_ of the lead peptide, TNF1, is added to the sum of the relative free energies of the individual point-variants required to generate TNF1-opt, a predicted K_d_ of 1.9–0.7 µM is obtained (Table 2). This matches well with the observed TNF1-opt K_d_ of 1.6±0.3 µM and suggests that the affinity enhancements contributed by each of the four point variations in TNF1-opt are acting nearly independently [22]. If the energetic contributions of point variations are additive, then a plot of the observed vs. predicted binding energies for the multiple variants should have a slope of 1 (Figure 5). The slope of the best-fit line for the variants tested is 0.97±0.01, very close to the expected value. Detailed predicted and observed values for each of the variants tested are given in the supporting materials (Table S1 in File S1) and in almost every case, nearly additive energetic contributions of the point variations are observed. The variant sequence that deviates most from the predicted value is the D4S+P5Y+M7K triple variant. In this case there are three substitutions in close proximity that could result in nearest neighbor interactions that alter the energetic picture [20]. Combining the S11K variant, a three-residue separation from the nearest substitution, with these three proximal substitutions contributes additively.

### Effect of TNF1 Additive Affinity Optimization on Binding Specificity

In order to compare relative binding specificity of TNF1 and corresponding variants, the peptides were spotted as a microarray and TNF-α binding was tested in the presence of *E.coli* cell lysate. Spotting the peptides as a microarray allows for comparison of TNF-α binding specificity across all variants in a single experiment. Preliminary microarray studies indicated that *E.coli* cell lysate serves as an excellent heterogeneous competitor (PEB, CWD and SAJ unpublished data). To minimize dye effects on binding, dual-color dye-swap experiments were performed with Alexa-555 labeled TNF-α and Alexa-647 labeled *E.coli* lysate, then repeated under the same conditions with Alexa-647 labeled TNF-α and Alexa-555 labeled *E.coli* lysate. These data were normalized and the TNF-α/*E.coli* binding intensity ratios for TNF1 and corresponding variants calculated.

Binding intensity ratios on the microarrays show some increased variant peptide binding to *E.coli* lysate relative to TNF1 (Table S2 in File S1). TNF1-opt, when spotted on a surface, binds both labeled TNF-α and labeled *E.coli* lysate with greater apparent affinity.

**Figure 6:**
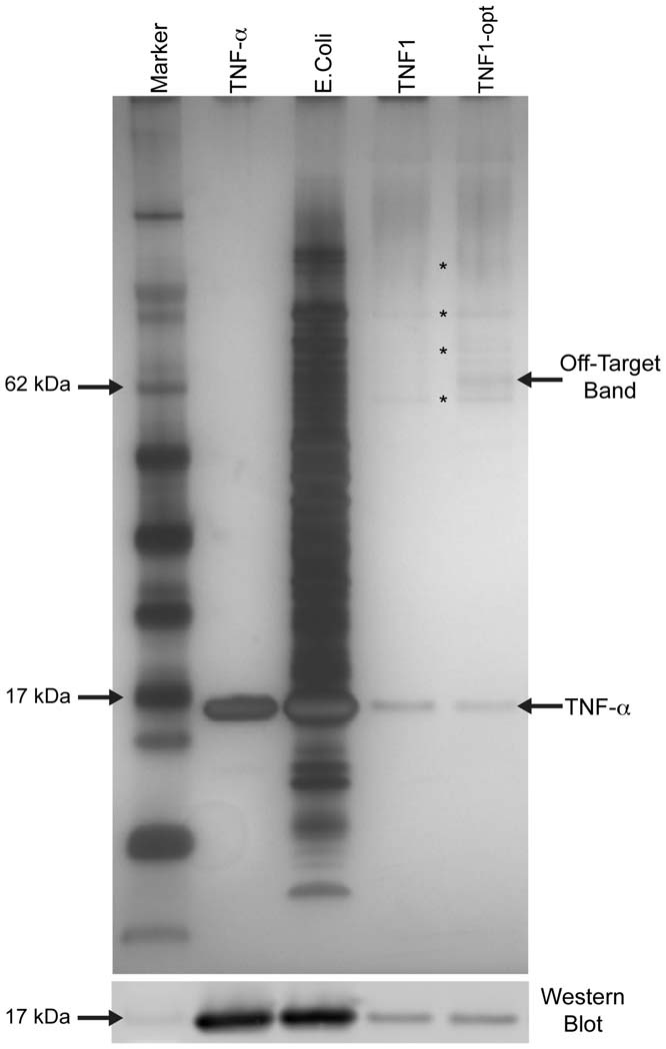
SDS-PAGE analysis of TNF1 and TNF1-opt pull-down assays. Silver-stained gel image of the final eluted fraction from TNF-α pull-down assays performed with immobilized TNF1 and TNF1-opt in the presence of excess *E.coli* lysate (∼2 mg/mL). Purified 10 µM TNF-α and the TNF-α spiked *E.coli* lysate used in the pull-down assay are also shown. Several bands appear on the gel independent of the immobilized peptide and are indicated with an asterisk (*), these bands are attributed to background bead binding. After subtracting background bands, one additional off-target band at approximately 65 kDa that appears in the TNF1-opt pull-down eluted fraction is noted. (Bottom) The band near 17 kDa in the SDS-PAGE image was validated as TNF-α with a Western blot.

Subsequent pull-down assays were performed with TNF1 and TNF1-opt immobilized on agarose beads in order to determine if the increased TNF1-opt *E.coli* lysate binding is due to general non-specificity or specific binding to a small number of *E.coli* proteins. TNF-α was spiked into an excess of *E.coli* lysate and incubated with the beads. SDS-PAGE analysis of the final eluted fraction shows that both TNF1 and TNF1-opt have good specificity for TNF-α (Figure 6). Only one additional band at approximately 65 kDa on the gel appears in the TNF1-opt eluted fraction when compared to TNF1. Several faint bands above and one below the 62 kDa marker appear in the gel, independent of the immobilized peptide, and are attributed to *E.coli* lysate binding to the beads (Figure 6). From this result, the increased TNF1-opt *E.coli* lysate binding observed on the microarray appears to be due primarily to enhanced binding to a single *E.coli* protein. Quantitative interpretation of this specificity data is somewhat difficult due to avidity on the surface, which can favor non-specific interactions [40].

### Additive Optimization of a Second Lead Peptide (TNF4) that Binds TNF-α

To test if this approach can be used to optimize lead peptides with a much better starting affinity, TNF4 (K_d_ = 23±3.5 µM for TNF-α) was optimized. From a screen of point-variants (Figure S8 in File S1), three affinity enhancing variations Y1W, D5Y and T10Y having the respective affinities K_d_ = 380±80 nM, K_d_ = 280±40 nM and K_d_ = 310±10 nM (Table S3 in File S1) were identified. From these point-variants, a double variant Y1W+D5Y with a K_d_ = 200±30 nM and a triple variant Y1W+D5Y+T10Y (referred to as TNF4-opt) with a K_d_ = 90±20 nM were produced (Table S3 in File S1). TNF4-opt has an approximate 250-fold enhancement in affinity relative to the TNF4 lead, which represents a larger affinity enhancement with three substitutions in TNF4-opt compared to four substitutions in TNF1-opt. This larger enhancement in TNF4-opt is thought to be due to the fact that the point-variants exhibit a more than 70-fold average enhancement relative to TNF4 (Table S3 in File S1) compared to a 3.4-fold average enhancement in the TNF1 point-variants (Table 1). TNF4-opt binding kinetic fits of SPR sensorgrams across several concentrations (Figure S9 in File S1) produce a k_off_ = 7.5±0.8×10^−3^ s^−1^ and k_on_ = 5.8±1.2×10^4^ M^−1^s^−1^, resulting in a K_d_ = 130±30 nM, which is in agreement with the affinity determined from equilibrium binding responses. TNF-α pull-down assays performed in the presence of excess *E.coli* lysate with TNF4 and TNF4-opt immobilized on beads indicate good TNF-α binding specificity (Figure S10 in File S1). After subtracting background bead binding, TNF4-opt produces one additional off-target band in the SDS-PAGE analysis when compared to TNF4.

### General Applicability of the Additivity Algorithm

To explore the generality of thermodynamic additivity for optimization of additional lead peptides and target proteins, affinity optimization of a peptide with the sequence AHKVVPQRQIRHAYNRYGSC (referred to as TRF26) known to bind weakly to the common blood protein transferrin (K_d_ = 85±14 µM) was performed. In this case, TRF26 was identified from a screen of the same initial library of 10^4^ random peptide sequences printed as a microarray on a glass slide (PEB, CWD and SAJ unpublished data), rather than high-throughput SPR screening as was done for TNF1 and TNF4.

A library of 323 TRF26 point-variants, substituting 19 of the 20 natural amino acids (excluding cysteine) in 17 positions of the peptide, was screened using array-based SPR with the peptides immobilized on a gold SPR chip surface (via a C-terminal cysteine) and 10 µM unlabelled transferrin in solution. The results of this point-variant screen are shown as a heat-map (Figure S11 in File S1). From this, two TRF26 point-variants (P6Y, H12F) were selected for further affinity characterization. The P6Y and H12F point-variants have dissociation constants of 8.6±1.6 µM and 9.7±1.6 µM respectively (Table S4 in File S1). Interestingly, a substitution set of 19 amino acids in the TRF26 point-variant screen did not produce proportionally more enhanced point variations than the 8 amino acid set used in the TNF1 or TNF4 point-variant screens, which suggests that a large amino acid substitution set is not required to identify affinity enhancing point variations. A TRF26 double variant sequence containing the P6Y+H12F variations was characterized by SPR. Assuming energetic additivity of point variations, the P6Y+H12F variant should have a K_d_ in the range of 1.3–0.7 µM. The observed P6Y+H12F variant K_d_ = 0.5±0.1 µM is in good agreement with this prediction (Table S5 in File S1).

## Discussion

We have utilized thermodynamic additivity of component variations to formulate a systematic algorithm for the development of peptide affinity reagents. This algorithm does not require large libraries or structural information, which are important characteristics that enable rapid, low-cost generation of high-affinity peptide ligands and differentiate this algorithm from other affinity reagent development approaches. Initially, a sparse sampling of random 20-mer peptide sequence space was screened for low-affinity lead peptides. After lead identification, the low-affinity peptides were optimized to high-affinity in only two steps by first screening restricted-diversity point-variant libraries, and second combining affinity-enhancing sequence variations to achieve thermodynamically additive binding affinity gains (Figure 1). General applicability to several protein targets has been shown, and binding dissociation constant (K_d_) enhancements of nearly 250-fold were achieved with this algorithm (Table S3 in File S1). In one specific example, the TNF4-opt peptide developed with this algorithm is one of the highest affinity TNF-α binding peptides/small-molecules reported to-date [28]–[30], [41].

While a comprehensive analysis of enthalpy/entropy contributions of variations in short unstructured peptides warrants a dedicated publication, this work provides insight into the effect of sequence variation on protein-peptide binding thermodynamics and specificity. First, in most cases tested, the effect of combining sequence variations was additive with respect to binding energy (Table 2, Tables S3, S5 in File S1). Exception to this was observed when the combined point-variations were nearest neighbors, in which case, the affinity gains were less than what would be expected from thermodynamic additivity. This deviation from additivity is presumably due to nearest neighbor interactions that interfere with independent contributions by each variation [17], [20]. It is also possible, in principle, for nearest neighbor interactions to result in cooperative gains, thereby improving affinity beyond what would be predicted by thermodynamic additivity, such cooperative interactions were not observed here.

Based on the lead peptides tested, it appears that higher lead peptide affinity produces higher affinity in an optimized sequence containing multiple ehancing point variations. Also, the somewhat less than additive affinity gains achieved with TNF4-opt, which was derived from the highest lead starting affinity (TNF4, K_d_ = 23±3.5 µM for TNF-α), suggest that additivity in a 20-mer peptide starts to break down as the affinity approaches the low nanomolar range (Table S3 in File S1). One explanation for this is that as additional contact points between the peptide and protein are added, the peptide becomes increasingly sterically constrained, making it impossible to add further contact points that are structurally independent [17], [20], [42]. A potentially powerful strategy to overcome this apparent affinity barrier is to combine this additivity algorithm with recently described multivalent peptide affinity reagent approaches [31], [43].

Both microarray-based and bead-based pull-down specificity studies show that TNF1-opt and TNF4-opt bind TNF-α with high specificity when challenged with *E.coli* lysate as a competive mixture (Figure 6, Figure S10 in File S1). Pull-down assays identified only one additional off-target *E.coli* protein bound by the optimized peptides when compared to the leads. This suggests that the additive affinity enhancements are not purely due to non-specific effects. Several possible explanations for the optimized variants binding to a single additional off-target protein exist. First the variations introduced into the optimized sequences could have produced a new binding motif in the peptides that have ‘specific’ affinity for a single *E.coli* protein. Second, it is possible *E.coli* protein(s) exist that contain binding surfaces(s) analogous to binding surface(s) on TNF-α and enhancing TNF-α binding site affinity also enhances affinity for analogous sites on *E.coli* protein(s). Finally, variant peptide binding to TNF-α may modify the surface of TNF-α in such a way that promotes an interaction between TNF-α and specific *E.coli* protein(s). Due to the distinct nature of binding affinity and specificity [44], [45], achieving significant gains in affinity as well as target specificity may be possible by simultaneously screening point-variants for both affinity and specificity enhancement before combining them into an optimized variant.

From the microarray binding experiments, it is worth noting that the ranked fluorescently labeled TNF-α binding intensities for TNF1 and all variants on the microarray (Table S2 in File S1) agree very well with the ranked affinities of the same peptides determined by SPR (Tables 1, 2). This demonstrates that short peptides screened and optimized in solution-phase assays such as SPR can show comparable target binding behavior when immobilized on a microarray surface [46], a potentially very useful characteristic when designing affinity reagents for purification or diagnostics.

The additivity algorithm described here provides several distinct advantages for affinity reagent development. First, without the use of enzymes to create the libraries, there is unbiased sequence selection/optimization [20], and this approach could be applied to natural/non-natural heteropolymers under a diverse range of conditions. Second, through judicious combination of point variations, specific properties of the final affinity reagent, such as solubility, tendency to aggregate, or performance in a particularly assay format, can be maintained or improved throughout the optimization process. Third, since this algorithm utilizes a screen, rather than a selection, both enhanced and reduced affinity variants can be quantified, providing significantly more information about the binding interaction when compared to a selection that only produces a small set of the highest affinity sequences. Finally, this approach uses a relatively small amount of target protein and is amenable to high-throughput application and automation, which is very important for producing a library of affinity reagents to the proteome. Because this is a systematic algorithm, one could envision an automated system that starts with a target protein, generates a lead sequence from a sparse random library, optimizes the peptide via thermodynamic additivity and outputs an optimized sequence.

## Methods

### Peptide SPR Screen

A library of 10^4^ 20-mer random sequence peptides was screened as a series of 4 experiments using a Biacore A100 (GE Healthcare, Piscataway, NJ) high-throughput surface plasmon resonance (SPR) system, with ∼2500 peptides screened in each experiment. Four peptides were flowed separately, in parallel, through four SPR flow cells with 5 proteins immobilized as addressable spots in each flow cell (a total of 20 addressable spots). Peptide binding was analyzed with a 60-second association phase followed by a 60-second dissociation phase. Reference subtracted SPR sensorgrams were recorded for each peptide at all protein spots, in all flow cells, on the SPR chip. Surface regeneration was performed after every 15 injections in each flow cell with Biacore Glycine 2.5 regeneration solution (GE Healthcare, Piscataway, NJ).

### Lead Identification

Lead peptides with TNF-α affinity were identified after a series of validation steps following the random peptide library screen. First, 171 potential lead peptides were screened for acceptable sample purity using MALDI-MS, those with purity less than 70% were discarded. The remaining potential lead peptides were further filtered by comparing TNF-α SPR binding response to the binding response from three unrelated proteins on the SPR chip as well as the response from the neutravidin coated reference spot. Peptides that showed significant binding response with any of the off-target proteins were discarded. Finally, the remaining 10 leads were subjected to a second SPR affinity assay using a series of peptide concentrations. From this, two lead peptides, TNF1 and TNF4, were identified.

### Point-Variant Library and SPR Chip Preparation

Point-variant libraries were prepared in 96-well stock plates as described in the supporting information. From the stock plate, peptides were diluted to 50 µM or 10 µM in Biacore HBS-EP buffer (GE Healthcare, Piscataway, NJ) containing 1 mg/ml carboxymethyl-dextran (Sigma-Aldrich, St. Louis, MO) to reduce non-specific binding to the CM-5 SPR chip surface. Biotinylated TNF-α was captured on a neutravidin coated Biacore CM-5 chip (GE Healthcare, Piscataway, NJ) at different levels on spots 1, 2, 4, and 5 across all four flow cells corresponding to a low to moderate R_max_ range of 40-200 RU. Spot 3 on all flow cells contained only immobilized neutravidin and served as a reference spot.

### Point-Variant SPR Screen

Using the prepared 96-well plates and Biacore A100 SPR system, four peptides were flowed separately, in parallel, through the four flow cells over all 4 TNF-α spots and the neutravidin reference spot (16 TNF-α, 4 neutravidin spots total), with a 60-second association phase and 300-second dissociation phase. Reference subtracted SPR sensorgrams were recorded for each peptide from all TNF-α spots. Surface regeneration was performed after every 12 injections in each flow cell with Biacore Glycine 2.5 regeneration solution. Point-variant reference subtracted, peptide molecular weight adjusted, responses at the late binding region of the sensorgram (a few seconds before dissociation) were compared to the response of the lead.

### Enhanced Point-Variant Characterization

Affinities of several enhanced point-variants identified from the point-variant screen were determined by SPR using equilibrium binding responses across a series of peptide concentrations on an SPR chip containing varying levels of immobilized TNF-α with a predicted R_max_ range of 40-120 RU. Responses were normalized to the predicted R_max_ so that results from different TNF-α capture levels could be directly compared.

### Multiple Variant Characterization

Sequences containing multiple enhancing point variations were synthesized using standard solid-phase synthesis and purified. Multiple variant affinities were determined with the same protocol used for point-variant affinities.

### Calculation of Binding Energies and Dissociation Constants

Peptide binding energies and dissociation constants (K_d_) were calculated separately as a mean and standard error of all replicate measurements. Therefore, the reported standard free energies of binding and dissociation constants may deviate slightly from direct calculation using the reported values. For multiple variants, predicted binding energies are reported as a range to account for the error in the observed values used to calculate the predicted value.

*Additional experimental details are available in the supporting information (Text S1 in File S1).*

## Supporting Information

File S1 Euclidian clustering of large-scale array data. Means of log_2_ transformed normalized TF activities from three replicates across three arrays were clustered. Data are denoted as described in Fig. S1. Replicates within arrays clustered together, indicating data could be blocked by array. (PDF)
