# Supplementary material for: Thermodynamic Additivity of Sequence Variations: An Algorithm for Creating High Affinity Peptides Without Large Libraries or Structural Information
Source: PLoS One. 2010 Nov 11;5(11):e15432. doi: 10.1371/journal.pone.0015432 (PMC2978705; doi:10.1371/journal.pone.0015432)
Supplement: File S1 — Euclidian clustering of large-scale array data. Means of log2 transformed normalized TF activities from three replicates across three arrays were clustered. Data are denoted as described in Fig. S1. Replicates within arrays clustered together, indicating data could be blocked by array. (PDF) [file pone.0015432.s001.pdf]

SUPPORTING INFORMATION

Thermodynamic Additivity of Sequence Variations: An Algorithm for Creating High Affinity Peptides Without Large Libraries or Structural Information

*Matthew P. Greving[1†], Paul E. Belcher[1], Chris W. Diehnelt[1], Maria J. Gonzalez-Moa[1], Jack Emery[1], Jinglin Fu[1], Stephen Albert Johnston[1,2], Neal W. Woodbury[1,3]**

Center for BioOptical Nanotechnology and Center for Innovations in Medicine, The Biodesign Institute[1], School of Life Sciences[2], Department of Chemistry and Biochemistry[3]

Arizona State University, Tempe, AZ 85287

E-mail: nwoodbury@asu.edu

*Address correspondence to: Neal W. Woodbury, 1001 S. McAllister Avenue, Tempe, AZ 85287-5201. Phone: 480-965-6671, Fax: 480-727-0396

[†]Current Address: The Scripps Research Institute, 10550 N. Torrey Pines Rd., La Jolla, CA 92037

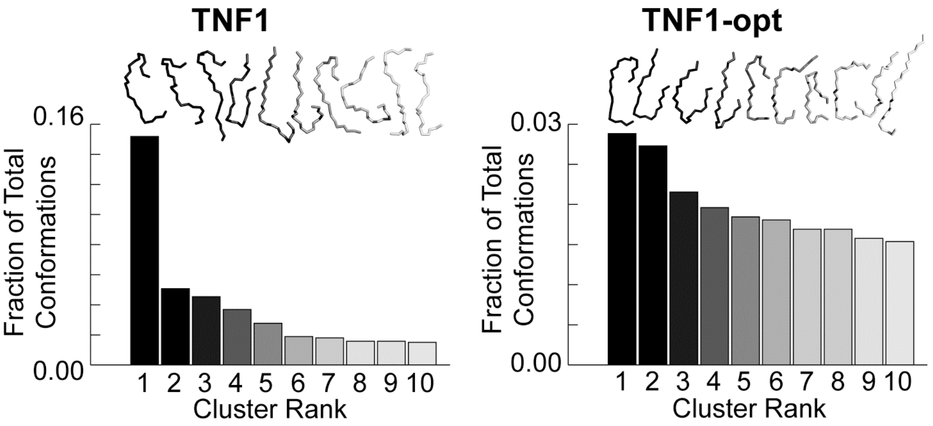

Figure S1. Molecular dynamics simulation of TNF1 and TNF1-opt peptide structure. Molecular dynamics (MD) simulations were performed to elucidate potential structure or structural tendencies in TNF1 and the effect of variations on possible conformations. In these simulations, 2600 sampled conformations from a total of 1 μs of MD trajectories were generated for both TNF1 and for TNF1-opt. These conformations were clustered by backbone structural alignment within 1 Å pair-wise RMSD. The fraction of the total number of conformations for the ten largest clusters is shown in the bar graph. Representative backbone conformations for the varied region of the peptide (residues 4-11) from each of the top ten clusters are shown above the graph, with the N-terminal end at the top and the structures ordered from cluster 1-10, left-to-right. Based on an analysis of the distribution of conformations, both peptides may be best characterized as loosely structured, with three main characteristics: 1) Both peptides have a tendency to form a loose and fluid hairpin, with the exact locus of the turn shifting among various positions in the region of residues 9-14. This is consistent with a negative band observed at 234 nm in their circular dichroism (CD) spectra [1,2,3] (Figures S2, S3); 2) The altered region of TNF1-opt, residues 4 through 11, substantially favored an extended conformation (though by no means rigid) in both TNF1 and TNF1-opt; and 3) apart from the foregoing, the structures of both peptides were quite flexible and variable overall.

While MD simulations suggest less rigidity in the TNF1-opt altered region, these simulations along with CD spectroscopy suggest that any tendency towards forming a hairpin present in TNF1 is retained in TNF1-opt. Similar structural tendencies in TNF1 and TNF1-opt imply that the four variations in TNF1-opt are not significantly structurally connected and therefore do not dramatically alter any structure or structural tendencies present in the lead peptide, supporting the idea that relatively unstructured peptides serve as good scaffolds for affinity optimization via thermodynamic additivity.

TNF1 Calculated Secondary Structure Fractions

Solutions from the CDSSTR method

Solutions using reference database: 7

Helix1	Helix2	Strand1	Strand2	Turns	Unordered	Total
0.33	0.08	0.17	0.11	0.09	0.22	1

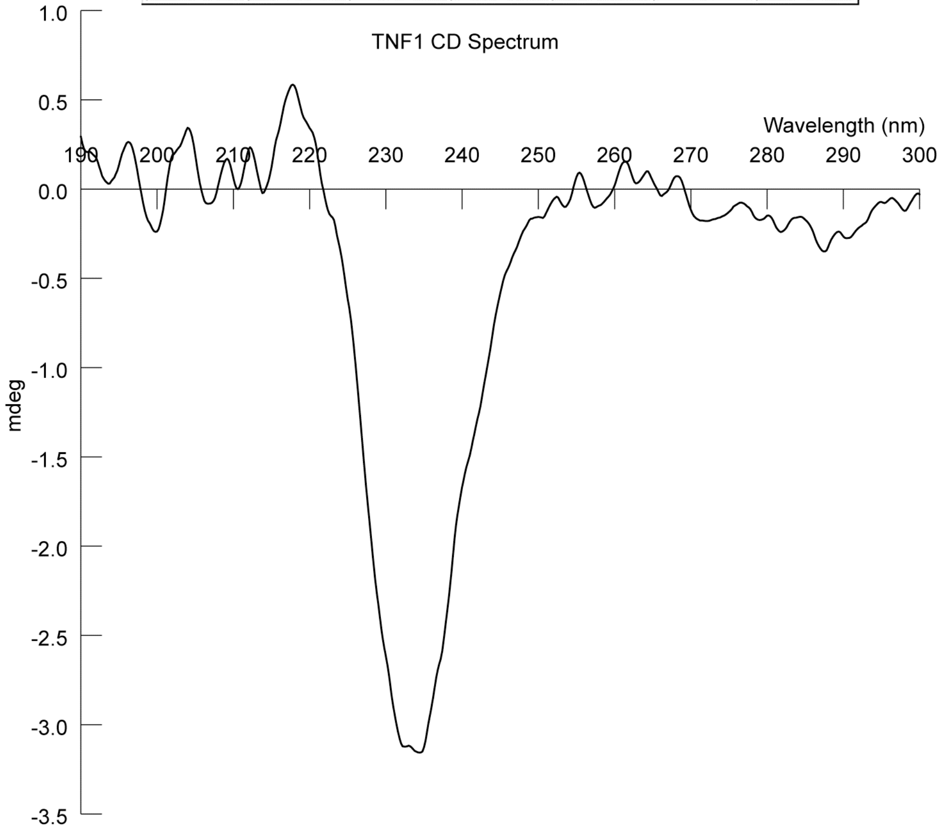

Figure S2. TNF1 CD spectrum and Dichroweb predicted secondary structure.

TNF1-opt

Calculated Secondary Structure Fractions

Solutions from the CDSSTR method

Solutions using reference database: 7

Helix1	Helix2	Strand1	Strand2	Turns	Unordered	Total
0.35	0.09	0.15	0.11	0.09	0.21	1

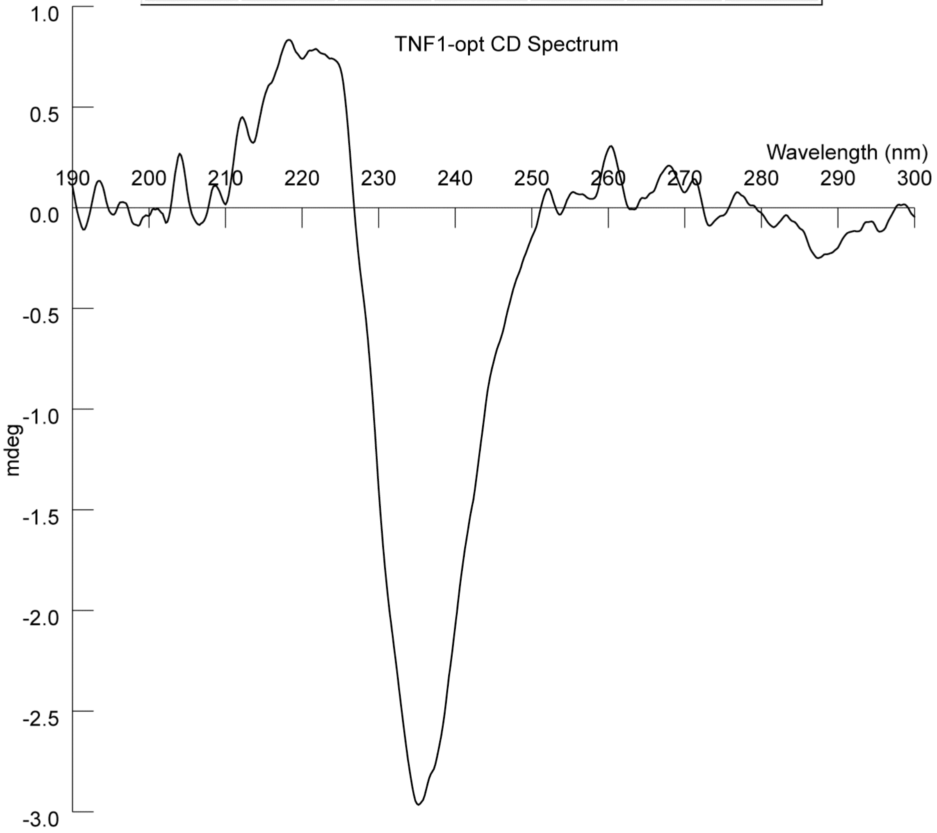

Figure S3. TNF1-opt CD spectrum and Dichroweb predicted secondary structure.

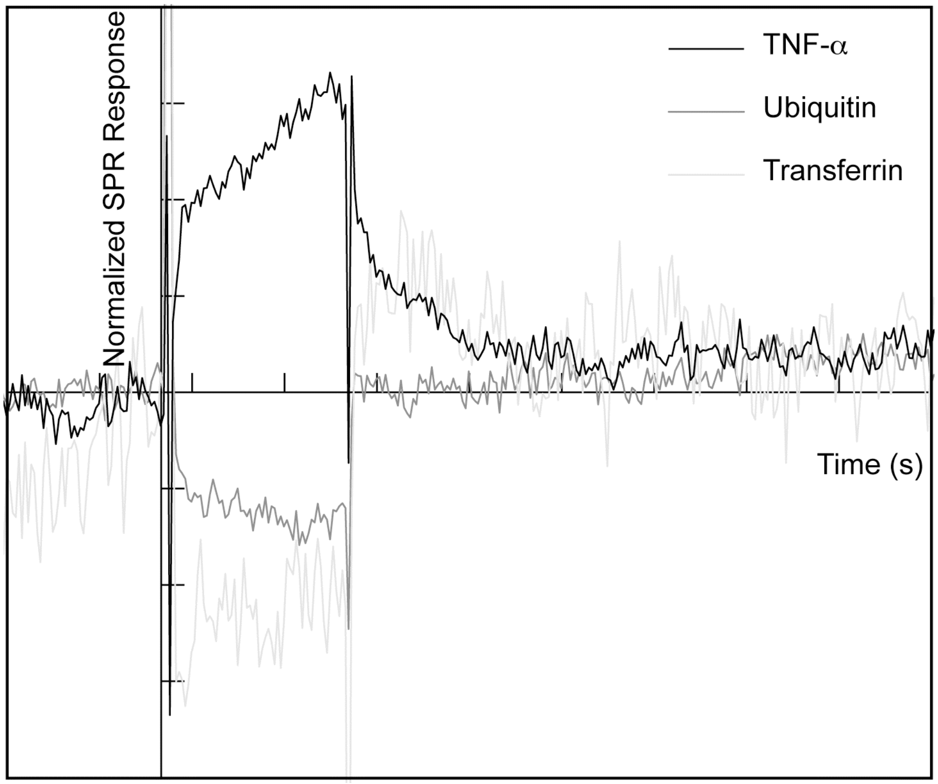

Figure S4. TNF1 lead peptide SPR response against a panel of five proteins. (TNF-α, Transferrin, Ubiquitin, AKT1 and Neutravidin). Relative SPR responses from the TNF1 lead peptide against immobilized TNF-α, Transferrin and Ubiquitin are shown. All measurements were taken on the same SPR chip with five different proteins immobilized on separate addressable spots on the SPR chip. Sensorgrams are normalized to the predicted maximum SPR response (R_{max}) for the specific protein molecular weight and immobilization level. TNF1 SPR responses against immobilized AKT1 and Neutravidin were also measured on the same chip as the three proteins shown. TNF1 showed no measurable binding response against AKT1 or Neutravidin, with AKT1 immobilized on an active spot and Neutravidin immobilized on the reference spot on the SPR chip. TNF1 peptide concentration was held constant at 50 μM for all measurements.

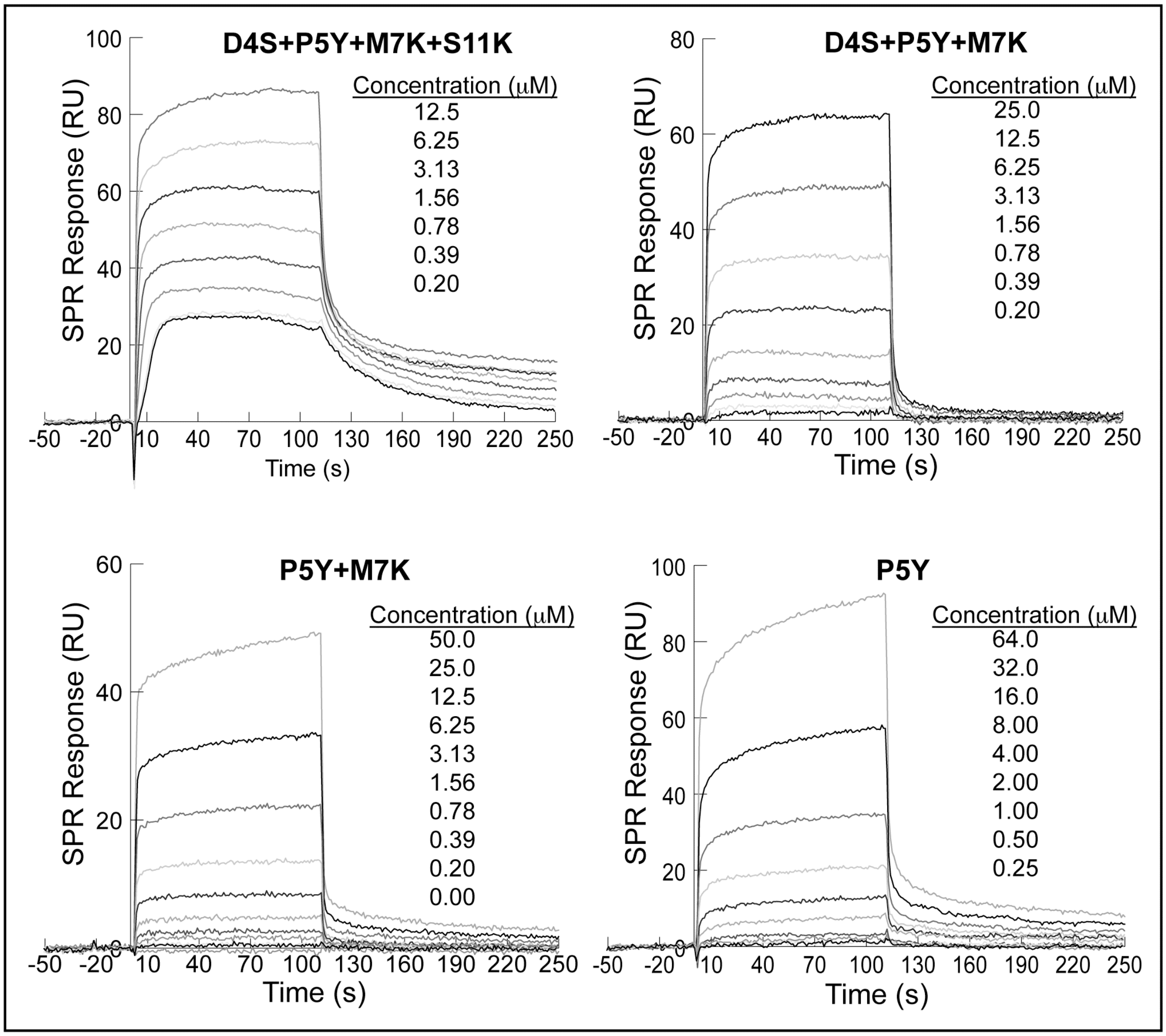

Figure S5. Typical sensorgrams for TNF1 quadruple (TNF1-opt), triple, double and single variant peptides.

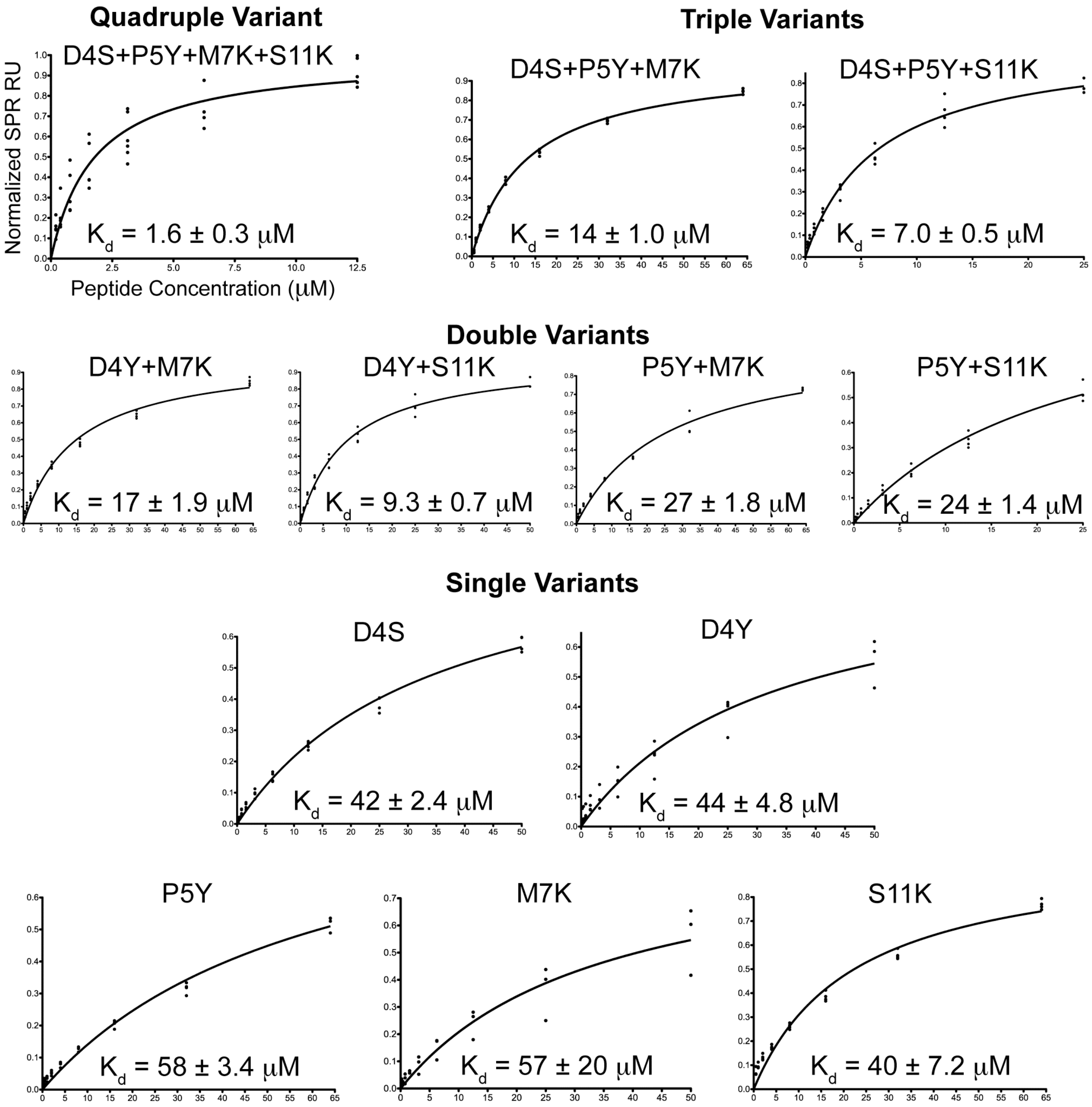

Figure S6. Binding isotherms for TNF1 single, double, triple and quadruple variant sequences. Data points correspond to the equilibrium SPR binding response from a particular peptide at a specific concentration.

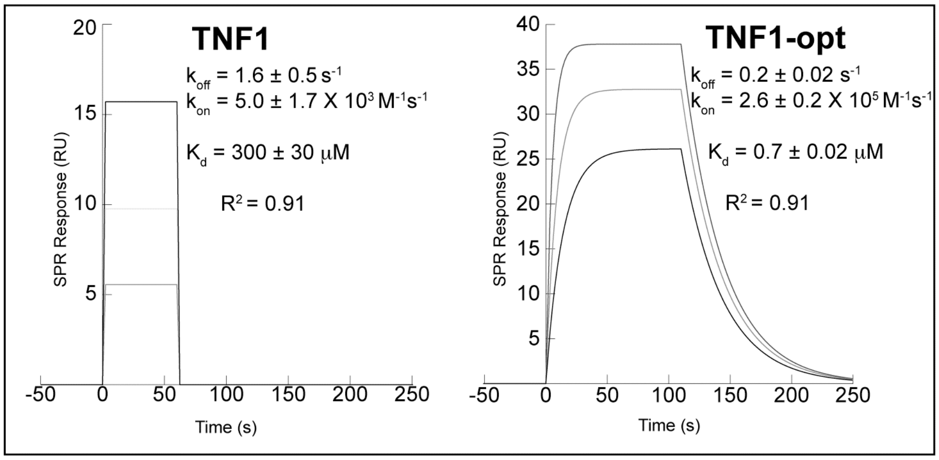

Figure S7. SPR sensorgram kinetic fits for TNF1 and TNF1-opt. On-rates (k_{on}), off-rates (k_{off}) and dissociation constants (K_d) shown were determined from kinetic fits of several sensorgrams across the same concentration series used in equilibrium binding response analysis.

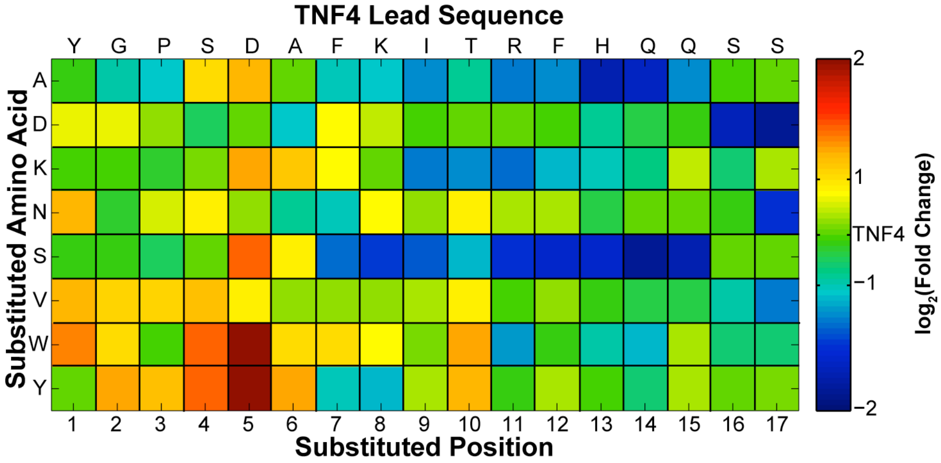

Figure S8. Fold-change heat map from the initial SPR screen of TNF4 point-variants. Fold-change relative to the TNF4 lead peptide was calculated from an average binding response after a 60-second association across several replicate injections of a fixed 10 μM peptide concentration.

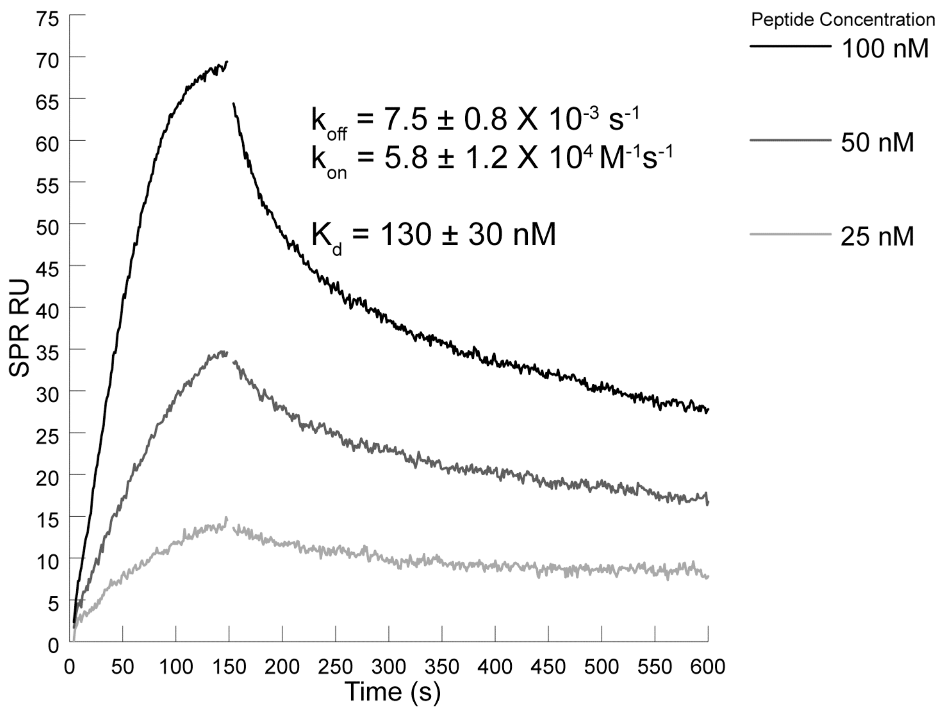

Figure S9. Typical sensorgrams for TNF4-opt.

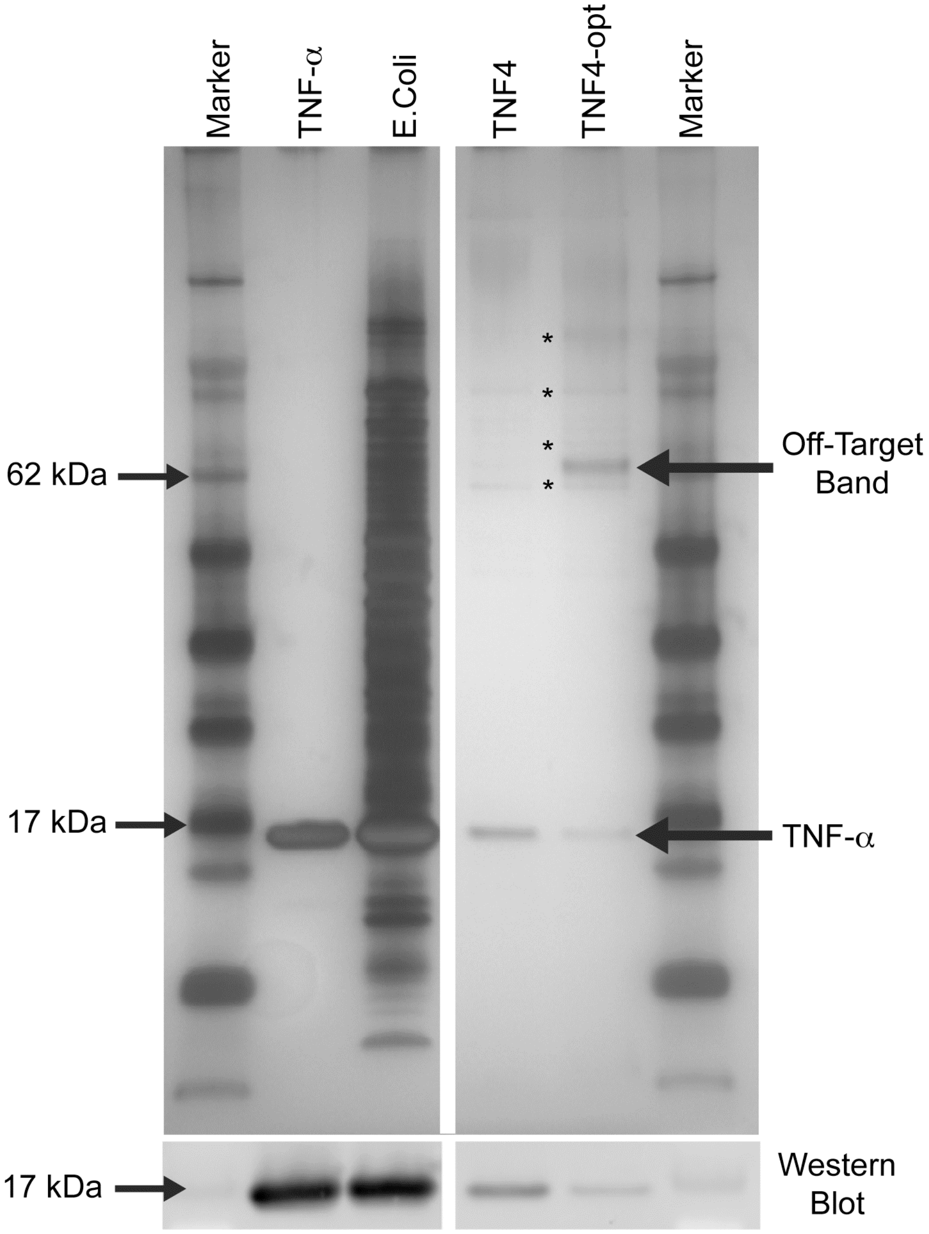

Figure S10. SDS-PAGE analysis of TNF4 and TNF4-opt pull-down assays. Silver-stained gel image of the final eluted fraction from TNF-α pull-down assays performed with immobilized TNF4 and TNF4-opt in the presence of excess *E.coli* lysate (~2 mg/mL). Purified 10 μM TNF-α and the TNF-α spiked *E.coli* lysate used in the pull-down assay are also shown. Several bands appear on the gel independent of the immobilized peptide and are indicated with an asterisk (*), these bands are attributed to background bead binding. After subtracting background bands, one additional off-target band at approximately 65 kDa that appears in the TNF4-opt pull-down eluted fraction is noted. (Bottom) The band near 17 kDa in the SDS-PAGE image was validated as TNF-α with a Western blot.

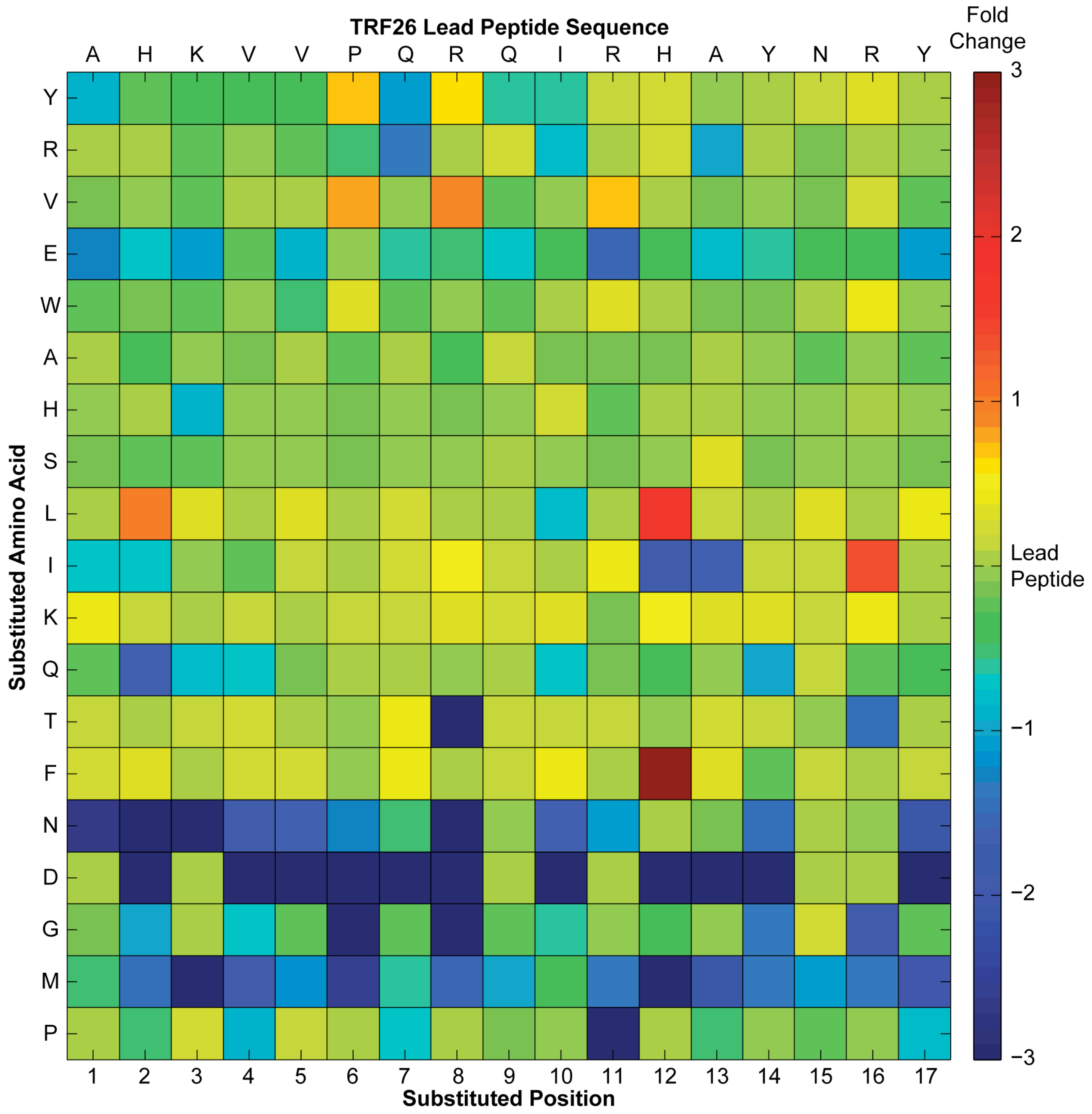

Figure S11. TRF26 point-variant library heat map. Point-variant peptides were immobilized on a gold SPR surface in an array format. The gold surface was blocked with BSA to reduce non-specific binding, and 10 μM transferrin was flowed over the SPR array. Fold-change color map corresponds to the equilibrium binding response of a particular point-variant relative to the TRF26 lead peptide.

Table S1. Observed standard free energies of binding and relative binding free energies for selected TNF1 multiple variant sequences differing by one or two component variations.[a]

**Peptide 1**	D4S+P5Y+ M7K	D4S+P5Y+ S11K	D4S+P5Y+ M7K+S11K	D4S+P5Y+ M7K+S11K	D4S+P5Y+ M7K+S11K	D4S+P5Y+ M7K+S11K
**Peptide 2**	P5Y+M7K	P5Y+S11K	D4S+P5Y+ M7K	D4S+P5Y+ S11K	P5Y+M7K	P5Y+S11K
**Peptide 1 Observed Standard Binding ΔG° (kcal/mol)**	-6.63 ± 0.04	-7.03 ± 0.04	-7.97 ± 0.11	-7.97 ± 0.11	-7.97 ± 0.11	-7.97 ± 0.11
**Peptide 2 Observed Standard Binding ΔG° (kcal/mol)**	-6.24 ± 0.04	-6.31 ± 0.04	-6.63 ± 0.04	-7.03 ± 0.04	-6.24 ± 0.04	-6.31 ± 0.04
**Peptide 1 - Peptide 2 Relative Binding ΔG (kcal/mol)**	-0.39 ± 0.06	-0.72 ± 0.06	-1.34 ± 0.12	-0.94 ± 0.12	-1.73 ± 0.12	-1.66 ± 0.12
**Peptide 1 and 2 Differential Component Variation(s)**	D4S	D4S	S11K	M7K	D4S, S11K	D4S, M7K

[a] The relative binding free energy differences observed between these variant peptides agrees well with the predicted difference calculated by assuming additivity and using the component contributions reported in Table 1 of the main text.

Table S2. Labeled TNF-α/E.Coli binding ratios and TNF-α binding intensities from dye-swap binding experiments performed with printed microarrays containing the TNF1 lead peptide and corresponding variants.

	**Microarray Results**		**Microarray Results Normalized to TNF1**	
Peptide (Lead and Variants)	Log2(TNF-α /E.Coli)	TNF-α Median Normalized Binding Intensity	(TNF-α/E.Coli) Ratio	TNF-α Binding Intensity
TNF1	1.21 ± 0.10	0.70 ± 0.12	1.00	1.00
D4S	0.53 ± 0.00	1.01 ± 0.18	0.63 ± 0.05	1.45 ± 0.36
P5Y	0.72 ± 0.03	1.01 ± 0.14	0.72 ± 0.07	1.44 ±0.31
M7K	0.76 ± 0.05	1.00 ± 0.19	0.73 ± 0.07	1.42 ± 0.36
S11K	-0.42 ± 0.09	0.94 ± 0.12	0.32 ± 0.07	1.34 ± 0.28
P5Y+M7K	0.33 ± 0.07	2.17 ± 0.31	0.55 ± 0.12	3.10 ± 0.69
P5Y+S11K	-0.26 ± 0.04	4.72 ± 0.47	0.36 ± 0.06	6.75 ± 1.33
D4S+P5Y+M7K	-0.22 ± 0.05	8.52 ± 0.87	0.37 ± 0.10	12.2 ± 2.41
D4S+P5Y+S11K	-0.16 ± 0.00	11.5 ± 0.74	0.39 ± 0.03	16.4 ± 2.97
D4S+P5Y+M7K+S11K	-0.18 ± 0.03	13.2 ± 4.50	0.38 ± 0.07	19.0 ± 7.20

Table S3. Observed standard free energies of binding and predicted relative binding free energies for selected TNF4 and corresponding variants.

	Peptide	TNF4	Y1W	D5Y	T10Y	Y1W+D5Y	Y1W+D5Y+T10Y
Observed	**Standard Binding ΔG° (kcal/mol)**	-6.37 ± 0.08	-8.80 ± 0.12	-8.98 ± 0.08	-8.87 ± 0.02	-9.14 ± 0.09	-9.68 ± 0.11
	**K_d (μM)**	**23 ± 3.5**	**0.38 ± 0.08**	**0.28 ± 0.04**	**0.31 ± 0.01**	**0.20 ± 0.03**	**0.09 ± 0.02**
	**K_d Fold-Change Relative to Lead**	-	61 ± 16	82 ± 18	74 ± 12	112 ± 24	244 ± 63
	**Variant Relative ΔG Contribution (kcal/mol)**	-	2.43 ± 0.14	2.61 ± 0.12	2.50 ± 0.09	-	-
Predicted	**Standard Binding ΔG° (kcal/mol)**	-	-	-	-	-11.4 ± 0.17	-13.9 ± 0.17
	**K_d Range (nM)**	**-**	**-**	**-**	**-**	**5.6 - 3.2**	**0.08 - 0.05**

Table S4. Observed standard free energies of binding and dissociation constants for the TRF26 lead peptide and point-variants selected from the point-variant library screen (Figure S11).

Peptide	TRF26 Lead	P6Y	H12F
**Standard Binding ΔG° (kcal/mol)**	-5.56 ± 0.10	-6.93 ± 0.11	-6.85 ± 0.06
**K_d (μM)**	**85 ± 14**	**8.6 ± 1.6**	**9.7 ± 1.6**
**K_d Fold-Change Relative to Lead**	-	10 ± 2.5	8.8 ± 2.0
**Variant Relative ΔG Contribution (kcal/mol)**	-	-1.37 ± 0.15	-1.29 ± 0.14

Table S5. Observed/Predicted standard free energies of binding and dissociation constants for the TRF26 P6Y+H12F double variant peptide.

	Peptide	P6Y + H12F
Observed	Standard Binding ΔG° (kcal/mol)	-8.68 ± 0.15
	K_d (μM)	**0.5 ± 0.1**
	K_d Fold-Change Relative to Lead	190 ± 57
Predicted	Standard Binding ΔG° (kcal/mol)	-8.22 ± 0.18
	K_d Range (nM)	**1.3 - 0.7**

Text S1: Experimental Details

Peptide Libraries. *Initial 10^4 Random Peptide Library.* Each 20-mer peptide was synthesized by Alta Biosciences (Birmingham, UK), based on the random sequences provided. Nineteen natural amino acids (excluding cysteine) were selected at random in each of the first 17 positions with Gly-Ser-Cys-COOH as the C-terminal linker. The synthesis scale was 2 µmole with about 70% purity and 2% of the peptides tested at random by mass spectrometry. Dry peptide was brought up in 100% dimethyl formamide until dissolved, then diluted 1:1 with purified water at pH 5.5 to a master concentration of 2 mg/ml. The original 96-deep-well plates were robotically transferred to 384-well plates, where the peptides were diluted to a ~1 mg/ml concentration in phosphate buffered saline at pH 7.2, before being further diluted 1:100 to give an SPR assay concentration of ~50 µM.

Point-Variant Libraries. Point-variant libraries were ordered in PEPScreen® 96-vial format from Sigma-Genosys (St. Louis, MO). The purity of each peptide in the library was assessed by MALDI-TOF mass spectrometry with typical sample purity ≥70%. Dry peptide was dissolved in HBS-N buffer to an approximate 2 mg/ml concentration, each vial was centrifuged to spin insoluble peptide to the bottom of the vial. Supernatant was collected from each vial and transferred to a 96-well plate, peptide supernatant concentrations in each well was measured using A_{280} absorbance and a predicted molar extinction coefficient (ε_{280}) based on the specific peptide sequence. Based on the supernatant concentrations, all the peptide wells were diluted to 250 µM concentration with HBS-EP buffer to make a stock plate(s). All SPR screens of the point-variants were done using dilutions from this stock plate(s).

SPR Assays. *SPR Chip Preparation.* Unless otherwise noted, SPR immobilization was done on a Biacore A100 SPR system (GE Healthcare, Piscataway, NJ). A standard coupling protocol was employed to immobilize a neutravidin capture surface via exposed primary amines. The immobilization was performed at 25 °C using Sodium Acetate (10 mM in 150 mM Sodium Chloride, pH 4.5) as the running buffer. Addressable spots 1, 2, 3 on each of the four flow cells of the CM-5 dextran SPR chip (GE Healthcare, Piscataway, NJ) were activated by a 10 minute injection of a freshly prepared 1:1 solution of 400 mM 1-ethyl-3-(3-dimethylaminopropyl)-carbodimide (EDC): 100 mM *N*-hydroxysuccimide (NHS) in water. The NHS-carboxy groups of spot 1 of the dextran surface were treated with a solution of neutravidin (50 µg/ml) in sodium acetate (pH 5.0) for 8 mins at a flow rate of 10 µl/min, residual active NHS groups were then quenched by a 5 min. pulse of ethylene diamine (1 M, pH 8.5). Similarly, neutravidin was coupled to spots 2 and 3 via a 12 and 16 min. injection cycle respectively, followed by quenching. An additional 7 min injection of ethylene diamine was then pulsed over spots 1-3 to ensure no activated sites remained. This immobilization process was then repeated to couple neutravidin to the carboxy terminus on addressable spots 4 and 5 to give a range of neutravidin immobilization levels across all 5 spots in each of the 4 flow cells.

Prior to the coupling of the biotin tagged protein, the surfaces of the SPR chip were conditioned with 8, 30-second pulses, of 10 mM hydrochloric acid. The biotinylated proteins were flowed over the respective spots at 10 µL/min until the desired level of capture was achieved, spot 3 in each of the 4 flow cells was used as a reference spot. Remaining unbound biotin binding sites on neutravidin were deactivated by two consecutive 30-second pulses of amino-PEG_2-Biotin (1 mM in HBS-N). Predicted R_{max} values based on protein capture levels for all assays were kept within the range of 40-200 RU.

TNF1 and TNF4 Lead Generation from a 10^4 Random Peptide Screen. *Peptide Library and SPR Chip Preparation.* Random 20-mer peptides were prepared in 384-well plates and diluted with Biacore HBS-EP buffer (GE Healthcare, Piscataway, NJ) containing 1 mg/ml carboxymethyl-dextran (Sigma-Aldrich, St. Louis, MO) to reduce non-specific binding to the CM-5 SPR chip surface. The final peptide concentration was 50 µM in each well. Peptides were then screened on a Biacore A100 SPR instrument (GE Healthcare, Piscataway, NJ). A CM-5 SPR chip (GE Healthcare, Piscataway, NJ) was prepared as described above with biotinylated Transferrin, TNF-α, Ubiquitin and AKT1 captured on spots 1, 2, 4 and 5 respectively on each of the four flow cells, Neutravidin was directly immobilized on spot 3 and used as a reference on each of the four flow cells.

TNF1-opt Fluorescence Polarization Affinity Characterization. Fluorescence anisotropy measurements were performed on a custom fluorimeter [4] with an orthogonal optical configuration. Two polarizers (Newman) were separately set before the excitation source and CCD array detector. TNF1-opt was labeled with 7-Diethylamino-3-(4-maleimidophenyl)-4-methylcoumarin (Sigma-Aldrich, St. Louis, MO), labeled peptide was isolated from free dye and unlabeled peptide using HPLC purification. For all measurements, TNF1-opt concentration was kept at 100 nM, TNF-α concentration was varied from 80 nM to 10 µM.

Binding Specificity on a Microarray. TNF1 and corresponding variants were spotted on aminopropyltriethoxysilane glass slides (made in-house) functionalized with Sulfo-SMCC (Thermo Fisher Scientific, Rockford, IL), leaving a reactive maleimide group free for covalent attachment via the C-terminal cysteine in the peptides. Peptides were diluted to 1 mg/ml in PBS and robotically spotted in triplicate in a humidity chamber and allowed to react overnight. Spotted slides were first washed with 90% TFA for 15 minutes, then rinsed with nanopure water and dried in a centrifuge. Spotted slides were then washed with 100 mM $NaBH_4$ in 2X SSC buffer containing 0.05% SDS for 15 minutes, rinsed with 1X SSC then PBST and finally with nanopure water and dried in a centrifuge. Microarrays were preblocked with 3% BSA (wt:wt) and 1 mM mercaptohexanol (to quench remaining maleimides) in PBST buffer for 2 hours in a humidity chamber then briefly rinsed with PBST. 200 nM TNF-α and 200 nM *E.coli* lysate (calculated as a total protein concentration in the lysate) labeled with Alexa-555 and Alexa-647 (Invitrogen, Carlsbad, CA) respectively in PBST buffer containing 1% BSA was incubated with the microarray for 1 hour in a humidity chamber. Dye-swap experiments were done in an identical manner with the exception of TNF-α and *E.coli* lysate being labeled with Alexa-647 and Alexa-555 respectively. After binding, the microarray was washed 3 times with PBST, 15 minutes each wash, and then 3 times with nanopure water, 15 minutes each wash. Finally the microarray was dried in a centrifuge and scanned with an Agilent (Santa Clara, CA) microarray scanner at 10 µm resolution and PMT settings adjusted to give similar dynamic range of intensities across both channels. Image analysis was done with Genepix (Molecular Devices, Sunnyvale, CA) and resulting data processed in Matlab (Natick, MA).

Binding Specificity Pull-Down Assays. 400 µL of SulfoLink® Coupling Resin slurry, equivalent to 200 µL of settled beads, (Thermo Scientific, Rockford, IL) was placed in a centrifuge column and washed 5x with coupling buffer (0.1 M phosphate buffer pH 8 containing 1 mM EDTA). Then, TNF1, TNF1-opt, TNF4 or TNF4-opt peptides dissolved in coupling buffer were immobilized on the resin by incubating 15 nmol of the corresponding peptide (200 µL of 75 µM solution) at room temperature for 1 hour. The resin was washed 3x with coupling buffer and the unreacted sites quenched with 400 µL of 100 mM mercaptohexanol in coupling buffer at room temperature for 45 min. After washing the

column 5x with 1M NaCl in 1x HBSN buffer, and 5x with 1x HBSN-T (1x HBSN containing 0.05% Tween-20), the resin is ready for the pull-down assay.

The peptide-bound resin was incubated with 100 µL of 10 µM TNF-α (PeproTech, Rocky Hill, NJ) in *E. coli* lysate (~2 mg/mL) for 1 hour at room temperature. The resin was washed consecutively, 5x with 1x HBS-NT, 3x with 1M NaCl in 1x HBS-N and 1x with 1x HBS-N. Finally, TNF-α was eluted by using 0.2 M NaOH at pH 9.0 and 100 µL fractions were collected. 75 µL of the first three fractions were combined and concentrated by centrifugation using a 3 kDa filter cut-off. Samples were run on a 4-12% SDS-PAGE gel (NuPAGE® Novex® Bis-Tris 4-12%, Invitrogen, Carlsbad, CA) for 10 min at 100 V and for 35 min more at 200 V and imaged using a Pierce Silver Stain Kit (Thermo Scientific, Rockford, IL). TNF-α was detected by Western Blot with an anti-TNF-α antibody (Invitrogen, Carlsbad, CD) and imaged on a Typhoon Trio (GE Healthcare, Piscataway, NJ).

TRF26 point-variant screen. The TRF26 lead peptide and point-variants were ordered in PEPScreen® 96-vial format from Sigma-Genosys and prepared as described above. From the 96-well stock plates, peptides were printed in microarray format on a bare gold Biacore FlexChip SPR (GE Healthcare, Piscataway, NJ) surface using a PerkinElmer (Waltham, MA) microarray spotter. Covalent attachment of the peptides was achieved through the C-terminal cysteine sulfhydryl group with the gold surface. After printing, the gold surface was treated with 1 mM β-mercaptoethanol to coat the remaining free gold surface with a monolayer of hydroxyl groups. β-mercaptoethanol treatment was done with the SPR chip in the Biacore FlexChip SPR system (GE Healthcare, Piscataway, NJ), allowing for real-time monitoring of monolayer attachment progress and completion. After monolayer attachment, the surface was washed extensively within the FlexChip system and 10 µM unlabelled transferrin in PBST buffer was flowed over the array surface. Binding response to each peptide spot was monitored and recorded over a 2 minute injection of transferrin. This experiment was repeated on a freshly prepared SPR array with the following additional step. After attachment of the β-mercaptoethanol monolayer, the gold surface was treated for 2 minutes with 1% Bovine Serum Albumin (BSA) in PBS to block non-specific binding. After BSA blocking, the surface was washed extensively and 10 µM unlabelled transferrin in PBST (Sigma-Aldrich, St. Louis, MO) was flowed over the SPR array for 2 minutes. The results from the both the unblocked and BSA blocked binding assays were comparable in that the point-variants with the highest binding responses were identical, however numerous peptides that showed moderate to low binding response in the unblocked assay showed dramatically reduced binding in the BSA blocked assay indicating that these moderate to low responses were likely non-specific.

TRF26 enhanced point-variant validation and characterization. TRF26 enhanced point-variants identified in the point-variant screen were synthesized and purified using standard solid-phase FMOC peptide synthesis and HPLC purification. Purified peptides were used for all validation and affinity characterization assays. Validation and affinity characterization of TRF26 point-variants was done using a Biacore T100 SPR instrument (GE Healthcare, Piscataway, NJ) and Biacore CM-5 SPR chips. CM-5 chip flow cells 1, 2, 3 and 4 were activated with EDC/NHS as described in the main article methods. After activation, 25 µg/ml transferrin in Acetate pH 4.5 buffer was flowed across flow cells 2 and 4 for 1000 seconds at 10 µl/min. Transferrin immobilization was adjusted to produce a predicted R_{max} ~ 100 RU. After transferrin immobilization, remaining active NHS groups were quenched with ethylene diamine on flow cells 1, 2, 3, and 4, with flow cells 1 and 3 serving as reference cells. Affinity characterization was done with a concentration series of unlabelled peptide flowed over the immobilized transferrin in HBS-P (GE Healthcare, Piscataway, NJ) buffer containing

1 mg/ml carboxymethyl-dextran (Sigma-Aldrich, St. Louis, MO) to inhibit non-specific binding to the CM-5 surface. Reference subtracted equilibrium binding response was recorded after a minimum 60-second association. Surface regeneration was done with Biacore Glycine 2.5 regeneration solution (GE Healthcare, Piscataway, NJ).

Molecular Dynamics Simulations. For each sequence, 100 molecular dynamics trajectories, each of 10 ns in length, were generated using AMBER v.9 [5]. Each trajectory was begun from a conformation generated by assigning random values to all rotatable bonds, then randomly rotating bonds to eliminate any steric collisions, then minimizing. Trajectories were run using a 2 fs time step, with bonds to hydrogens constrained with SHAKE [6], AmberParm96 force field parameters, and the GB/SA implicit solvent model, with parameter settings SALTCON = 0.15, SURFTEN = 0.003, and EXTDIEL = 75 to simulate the salt, surfactant, and organic content of the SPR running buffer used for affinity measurements. Temperature for all runs was maintained at 300K via the Andersen thermostat [7] applied at 4 ps intervals. Conformations were sampled at 200 ps intervals after discarding the first 5 ns of each trajectory, yielding a total of 2600 samples for each sequence. A 2600 x 2600 pairwise distance matrix was computed reflecting average RMS distances following structural alignment of the backbone atoms of residues 4 through 11, as computed for each pair of conformations using Pymol's [8] fit function. Clustering was performed by repeatedly identifying the largest subset of samples having RMS distances within a 1 Å threshold, and removing the cluster so identified from the distance matrix. The graphical representations in Figure S1 were produced using Pymol.

TNF1 and TNF1-opt Circular Dichroism Spectroscopy. All circular dichroism (CD) spectroscopy was done using a JASCO J-815 CD (Easton, MD). TNF1 and TNF1-opt peptides were diluted to 10 µM in HBS buffer with 0.015% P-20 detergent. A blank 190-300 nm CD spectrum was taken before all measurements with HBS 0.015% P-20 buffer; this blank spectrum was subtracted from all subsequent peptide spectra. CD spectra for both TNF1 and TNF1-opt between 190-300 nm were taken at 25 °C.

SUPPORTING REFERENCES

1. Fasman GD (1996) Circular Dichroism and the Conformational Analysis of Biomolecules. New York: Plenum Press.
2. Rana S, Kundu B, Durani S (2005) A small peptide stereochemically customized as a globular fold with a molecular cleft. Chem Commun: 207-209.
3. Roy RS, Gopi HN, Raghothama S, Gilardi RD, Karle IL, et al. (2005) Peptide hairpins with strand segments containing alpha- and beta-amino acid residues: cross-strand aromatic interactions of facing Phe residues. Biopolymers 80: 787-799.
4. Kelbauskas L, Chan N, Bash R, Yodh J, Woodbury N, et al. (2007) Sequence-dependent nucleosome structure and stability variations detected by Förster resonance energy transfer. Biochemistry 46: 2239-2248.
5. Case DA, Darden TA, Cheatham I, T.E. , Simmerling CL, Wang J, et al. (2006) AMBER 9. San Francisco: University of California.
6. Ryckaert JP, Ciccotti G, Berendsen HJC (1977) Numerical Integration of the Cartesian Equations of Motion of a System with Constraints: Molecular Dynamics of N-Alkanes. J Comput Phys 23: 327-341.
7. Andrea TA, Swope WC, Andersen HC (1983) The role of long ranged forces in determining the structure and properties of liquid water. J Chem Phys 79: 4576-4584.
8. DeLano WL (2008) The PyMOL Molecular Graphics System. Palo Alto, California, USA: DeLano Scientific.
